# The Role of the Plasma Membrane H^+^-ATPase in Plant Responses to Aluminum Toxicity

**DOI:** 10.3389/fpls.2017.01757

**Published:** 2017-10-17

**Authors:** Jiarong Zhang, Jian Wei, Dongxu Li, Xiangying Kong, Zed Rengel, Limei Chen, Ye Yang, Xiuming Cui, Qi Chen

**Affiliations:** ^1^Faculty of Life Science and Technology, Kunming University of Science and Technology, Kunming, China; ^2^Faculty of Architecture and City Planning, Kunming University of Science and Technology, Kunming, China; ^3^UWA School of Agriculture and Environment, Faculty of Science, University of Western Australia, Perth, WA, Australia

**Keywords:** Al toxicity, H^+^-ATPase, Mg, IAA, transgenic, citrate exudation

## Abstract

Aluminum (Al) toxicity is a key factor limiting plant growth and crop production on acid soils. Increasing the plant Al-detoxification capacity and/or breeding Al-resistant cultivars are a cost-effective strategy to support crop growth on acidic soils. The plasma membrane H^+^-ATPase plays a central role in all plant physiological processes. Changes in the activity of the plasma membrane H^+^-ATPase through regulating the expression and phosphorylation of this enzyme are also involved in many plant responses to Al toxicity. The plasma membrane H^+^-ATPase mediated H^+^ influx may be associated with the maintenance of cytosolic pH and the plasma membrane gradients as well as Al-induced citrate efflux mediated by a H^+^-ATPase-coupled MATE co-transport system. In particular, modulating the activity of plasma membrane H^+^-ATPase through application of its activators (e.g., magnesium or IAA) or using transgenics has effectively enhanced plant resistance to Al stress in several species. In this review, we critically assess the available knowledge on the role of the plasma membrane H^+^-ATPase in plant responses to Al stress, incorporating physiological and molecular aspects.

## Introduction

Acid soils (pH_water_ < 5.5) are found worldwide, occupying up to 50% of arable land (Kochian et al., 2015), mostly in developing countries in Africa, Asia, and South America. Aluminum (Al) toxicity is a major factor limiting plant growth and crop yield in acidic soils. Al toxicity has been shown to affect the plasma membrane structure, induce root cell death and inhibit nutrient uptake, thereby leading to significant reductions in plant growth and development (Chen et al., 2011). Liming can increase soil pH, but this is frequently neither an economic option for farmers nor an effective strategy for alleviating subsoil acidity (Whitten et al., 2000). In contrast, increasing capacity of plants to cope with Al toxicity by enhancing Al detoxification and/or breeding Al-resistant cultivars is a more cost-effective way of mitigating the problem.

The plasma membrane H^+^-ATPase, the most abundant membrane protein as a single polypeptide of about 100 kDa, belongs to a large superfamily of pumps termed P-type ATPases (Palmgren, 2001; Rengel et al., 2015). Using the chemical energy of ATP, the plasma membrane H^+^-ATPases extrude protons from cells to generate an electrochemical proton gradient. The generation of this gradient has a major role in providing the energy for secondary ion transport across the plasma membrane, enabling physiological functions such as nutrient uptake, intracellular pH regulation, stomatal opening, and cell growth (Falhof et al., 2016). Additionally, the plasma membrane H^+^-ATPase is involved in plant adaptation to environmental stresses, such as salinity, P deficiency and Al toxicity (Janicka-Russak and Kabala, 2015; Yu et al., 2015).

Aluminum toxicity affects the expression and post-translation of the plasma membrane H^+^-ATPase in some plant species (Shen et al., 2005; Chen et al., 2013, 2015; Guo et al., 2013). In particular, modulation of the activity of this enzyme through application of Mg, IAA or using transgenic methods can effectively enhance Al-resistance in faba bean (Chen et al., 2015), soybean (Wang et al., 2016), *Arabidopsis* (Wu et al., 2008), and tobacco (Guo, 2013). The plant Al-resistance mechanisms, especially those based on Al-induced organic acid anion exudation, have been reviewed many times (Liu et al., 2014; Kochian et al., 2015; Chen and Liao, 2016); however, a role of the plasma membrane H^+^-ATPase in plant responses to Al stress has not been well assessed. In this review, we appraise the literature on this nascent field, paying particular attention to physiological and molecular aspects of the plasma membrane H^+^-ATPase involvement in plant responses to Al stress.

## Aluminum Detoxification Strategies

Plants have evolved two main Al resistance strategies, namely avoidance and tolerance. Tolerant plants, such as buckwheat (Ma et al., 1998), hydrangea (Ma et al., 1997), melastoma (Watanabe et al., 1998), and tea (Morita et al., 2008) allow Al accumulation in plant tissues, using Al sequestration in the vacuole and/or Al detoxification via Al binding to organic acid anions or proteins as the tolerance mechanisms. In contrast, plants with the avoidance mechanisms decrease Al accumulation in roots via cell wall polysaccharide modifications (Schmohl et al., 2000; Yang et al., 2008, 2011a, 2016; Li et al., 2016) or exudation of organic acid anions from root tips (Ma, 2000; Chen and Liao, 2016).

Many publications progressed the knowledge of plant resistance to Al stress (Kochian et al., 2015; Chen and Liao, 2016). For example, Al resistance is associated with upregulation of the ABC transporters (*ALS3* and *ALS1*) in *Arabidopsis* (Larsen et al., 2005, 2007; Huang et al., 2009) and the bacterial-type ABC transporters (*STAR1* and *STAR2*) in rice (Huang et al., 2009). The Al-induced expression of the *ALMT* (Al-activated malate transporter) and *MATE* (multidrug and toxic compound extrusion) transporter genes (Sasaki et al., 2004; Hoekenga et al., 2006; Magalhaes et al., 2007) enhances root exudation of malate and citrate, respectively. The zinc-finger transcription factors, such as STOP1 (sensitive to proton rhizotoxicity) in *Arabidopsis* (Iuchi et al., 2007; Sawaki et al., 2009) and ART1 (Al resistance transcription factor) in rice (Yamaji et al., 2009), play central roles in regulating expression of many genes associated with Al resistance. Additionally, activation of the plasma membrane H^+^-ATPase through regulating the expression and/or phosphorylation of this enzyme coincides with citrate exudation and Al resistance in many plant species (Yu et al., 2015).

## Functions and Regulation of the Plasma Membrane H^+^-ATPase

### Critical Functions

**FIGURE 1:**
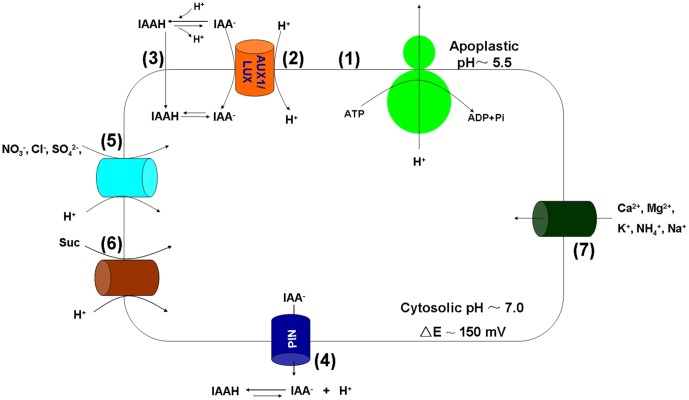
The functions of the plasma membrane H^+^-ATPase in IAA polar transport and nutrient uptake. (1) The proton pump; (2) AUX1/LAX-mediated IAA^-^/H^+^ symport; (3) Lipophilic diffusion of protonated IAAH; (4) PIN proteins-mediated IAA^-^ efflux; (5) Anion (NO_3_^-^, Cl^-^, SO_4_^2-^, etc.) uptake via symport with H^+^; (6) Proton-coupled sucrose transporters-mediated sucrose (Suc) phloem loading; (7) cation (Mg^2+^, Ca^2+^, K^+^, etc.) uptake through various transporters.

The plasma membrane H^+^-ATPases (proton pumps) are the primary transporters that translocate positive charges (protons) out of the cytosol using ATP as an energy source, thereby forming a membrane potential difference across the plasma membrane (negative on the inside). Using the proton electrical gradients created by the plasma membrane H^+^-ATPase, cations (e.g., K^+^, Na^+^, NH_4_^+^, Mg^2+^, Ca^2+^), anions (e.g., NO_3_^-^, SO_4_^2-^, Cl^-^), and neutral compounds (e.g., glucose) can be taken up across the plasma membrane via secondary carrier proteins (**Figure 1**).

One of the most basic functions of the plasma membrane H^+^-ATPase in plants is the involvement in auxin polar transport and signaling. Auxin is synthesized in the plant apical growing regions and transported to roots through the vascular and bundle sheath tissues (Titapiwatanakun and Murphy, 2009). As protons accumulate in the apoplast (pH∼5.5) (lower pH than in the cytosol, **Figure 1**), 10–25% of IAA (pKa 4.85) is protonated to form electrically neutral IAAH (Yang et al., 2006). IAA can thus enter the cell by either lipophilic diffusion of protonated IAAH along the concentration gradient or via the AUX1/LAX influx carrier-mediated IAA^-^/H^+^ symport (**Figure 1**). Inside the cell, all IAA molecules are deprotonated because the cytosolic pH is relatively high (pH∼7.0). The efflux of IAA^-^ is accomplished by PIN proteins, resulting in activation of the plasma membrane H^+^-ATPase (**Figure 1**) and acidification of the cell wall (apoplast). For instance, activation of the plasma membrane H^+^-ATPase is important in auxin-mediated cell elongation during wheat embryo development (Rober-Kleber et al., 2003). In *Arabidopsis*, auxin induces hypocotyl elongation through phosphorylation and activation of the plasma membrane H^+^-ATPase (Takahashi et al., 2012).

### Regulatory Mechanisms

The plasma membrane H^+^-ATPase is encoded by a multigene family and contains several isoforms. For example, 11 isoforms of plasma membrane H^+^-ATPase in *Arabidopsis thaliana* (AHA1–AHA11) (Palmgren, 2001), 10 genes in *Oryza sativa* (OsA1–OsA10) (Arango et al., 2003), 9 genes in *Nicotiana plumbaginifolia* (PMA1–PMA9) (Oufattole et al., 2000), and 5 genes in *Vicia faba* (VHA1–VHA5) (Neuhaus et al., 2013) have been identified. Various genes are expressed in the same organ or plant tissues. For example, AHA1 and AHA2 are expressed in virtually all tissues and organs, AHA10 has been identified during seed development (Harper et al., 1994), and isoform AHA4 is expressed in the root endodermis, flowers and during silique maturation (Vitart et al., 2001). In faba bean, VHA1 and VHA2 are expressed mainly in guard cells, but to some extent throughout the plant as well (Hentzen et al., 1996). The expression of genes encoding the plasma membrane H^+^-ATPase is regulated by various environmental factors, including iron deficiency (Santi et al., 2005), Al toxicity (Yu et al., 2015), and heavy metals stress (Jemo et al., 2007).

The activity of H^+^-ATPase is tightly regulated by the phosphorylation and dephosphorylation processes, although neither the protein kinase nor the protein phosphatase directly regulating phosphorylation of this enzyme in plant cells has been identified. The best characterized mechanism involves the phosphorylation of the penultimate Thr residue in the autoinhibitory domain at the C-terminus, and the subsequent binding of 14-3-3 regulatory proteins (Falhof et al., 2016). The binding of 14-3-3 protein displaces the C-autoinhibitory domain, resulting in the formation of a dodecameric complex composed of six H^+^-ATPases and six 14-3-3 regulatory proteins and resulting in the activated plasma membrane H^+^-ATPase (Haruta et al., 2015). Many signals, including auxin, blue light and the fungal toxin fusicoccin (FC), regulate the phosphorylation level of the penultimate Thr at the C terminus of H^+^-ATPase and its interaction with the 14-3-3 proteins, affecting cell growth and stomatal movements (Emi et al., 2001; Hohm et al., 2014; Falhof et al., 2016; Wang et al., 2016).

## The Plasma Membrane H^+^-TPase and Aluminum Toxicity

### Changes in the Plasma Membrane H^+^-ATPase Activity in Plant Responses to Aluminum Toxicity

Under Al stress, the most important functional change occurring in the plasma membrane is the alteration of membrane potential (Ahn and Matsumoto, 2006), which is dependent on the plasma membrane H^+^-ATPase. The changes in the membrane potential and the plasma membrane H^+^-ATPase activity are related to the root part, the degree of Al stress and Al sensitivity of plant species and genotypes. For example, the Al stress induced depolarization of the plasma membrane in tobacco suspension-cultured cells (Sivaguru et al., 2005), squash (Ahn et al., 2001), and barley (Matsumoto, 1988), suggesting decreased H^+^-ATPase activity, but either depolarization or hyperpolarization of the plasma membrane was recorded in *Arabidopsis* (Bose et al., 2010a,b) and soybean (Wang et al., 2016). In wheat root tips, Al exposure caused hyperpolarization in the Al-resistant and depolarization in the Al-sensitive genotypes (Ahn et al., 2004). Al-induced proton release is associated with activation of the plasma membrane H^+^-ATPase in tea plants (Qing et al., 2017). In soybean and faba bean roots, Al significantly increased the activity of the plasma membrane H^+^-ATPase in the Al-resistant but not the Al-sensitive cultivars (Shen et al., 2005; Kim et al., 2010; Chen et al., 2013).

The activity of plasma membrane H^+^-ATPase is affected by Al at the transcription, translation, and post-translation levels. In soybean and faba bean, higher plasma membrane H^+^-ATPase concentration and activity coincided with enhanced expression of the gene and greater abundance of the plasma membrane H^+^-ATPase protein in the Al-resistant cultivars (Shen et al., 2005; Guo et al., 2013). Furthermore, Al increased the phosphorylation levels of the plasma membrane and its interaction with the 14-3-3 proteins in a time-dependent manner in the Al-resistant cultivars of faba bean and soybean (Shen et al., 2005; Chen Q. et al., 2012; Chen et al., 2013). It is likely that Al activates an unknown kinase, resulting in phosphorylation of the plasma membrane H^+^-ATPase, maintenance of strong interaction with the 14-3-3 proteins and thus increased H^+^-ATPase activity. However, identification and characterization of a kinase that regulates phosphorylation of the plasma membrane H^+^-ATPase remains to be reported.

### Involvement of the Plasma Membrane H^+^-ATPase in MATE-Mediated Citrate Exudation

Citrate and malate are intermediates in the tricarboxylic acid (TCA) cycle. The Al-induced organic acid anion exudation is controlled by specific transporters, such as ALMT1 for malate and MATE for citrate, along with enzymes of organic acid metabolism, such as citrate synthase, malate dehydrogenase, and phosphoenolpyruvate carboxylase (Chen and Liao, 2017). Beside the expression of citrate transporter, the exudation of citrate also requires activation of the plasma membrane H^+^-ATPase as shown in many plant species. Under low-P conditions, increased citrate exudation was related to activation of the plasma membrane H^+^-ATPase in white lupin (*Lupinus albus*) (Yan et al., 2002) and blue lupin (*Lupinus pilosus*) (Ligaba et al., 2004). A mutant carrot (*Daucus carota*) cell line (can grow on water-insoluble phosphate) exhibited an enhancement in citrate exudation and plasma membrane H^+^-ATPase activity when grown in an Al-phosphate medium (Ohno et al., 2003). In Al-resistant soybean roots under Al stress, fusicoccin significantly enhanced (by 85%) and vanadium ions significantly inhibited (by 53%) activity of the plasma membrane H^+^-ATPase, which was associated with Al-induced citrate exudation increasing by 58% and decreasing by 52% in the fusicoccin and vanadium treatments, respectively (Shen et al., 2005). Chen et al. (2013) in faba bean and Guo et al. (2013) in soybean reported similar results.

In soybean roots, the Al-induced enhancement of the plasma membrane H^+^-ATPase and the related citrate exudation coincided with the increased gene expression and protein abundance as well as enhanced phosphorylation of this enzyme (Shen et al., 2005; Guo et al., 2013). In both Al-resistant and Al-sensitive cultivars of faba bean, the interaction between the phosphorylated plasma membrane H^+^-ATPase and the 14-3-3 protein was stimulated by FC but inhibited by 5′-AMP (adenosine 5′-monophosphate) in the presence of Al; in addition, the activity of the plasma membrane H^+^-ATPase and the related citrate exudation were increased, respectively, about 1.7- and 2.7-fold by FC and decreased, respectively, about 60 and 70% by 5′-AMP (Chen et al., 2013), indicating that post-translational regulation of the plasma membrane H^+^-ATPase plays an important role in Al-induced citrate exudation.

It is interesting to note that, unlike the secretion of malate and oxalate, only the exudation of citrate is dependent on the plasma membrane H^+^-ATPase. For example, neither P-deficiency-induced malate exudation by *Lupinus pilosus* (Ligaba et al., 2004) nor Al-induced oxalate exudation by tomato (Yang et al., 2011b) was related to the plasma membrane H^+^-ATPase activity. Wu et al. (2014) found that transgenic *Arabidopsis* lines containing *Brassica oleracea* MATE gene had stronger citrate exudation coupled with a higher H^+^ efflux activity than wild-type plants.

**FIGURE 2:**
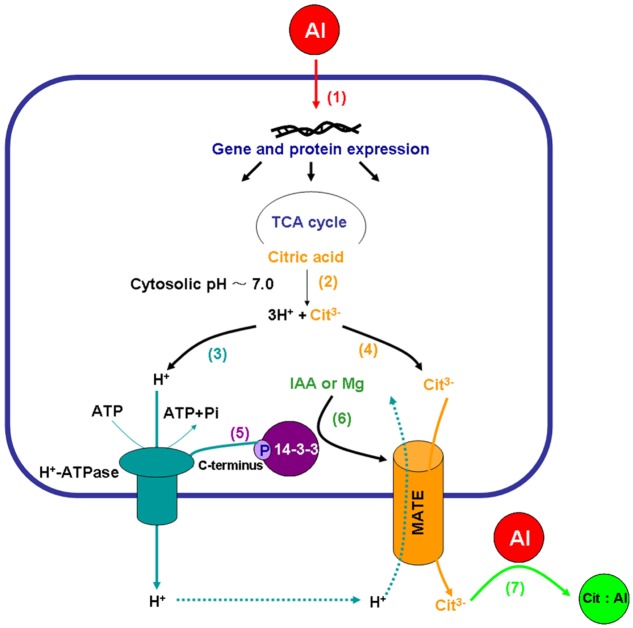
The proposed regulatory roles of plasma membrane H^+^-ATPase in Al-induced citrate exudation from plant roots. Al toxicity is perceived by root cells, inducing expression of numerous genes and proteins (1), including tricarboxylic acid (TCA) cycle enzymes, the plasma membrane H^+^-ATPase, 14-3-3 proteins and MATE transporters. Citric acid is dissociated to citrate anion (Cit^3-^) and H^+^ at a relative high cytosolic pH∼7.0 (2), followed by the plasma membrane H^+^-ATPase-mediated H^+^ efflux (3) coupled with MATE-mediated citrate (Cit^3-^) exudation (4) possibly associated with proton influx. The phosphorylation of the plasma membrane H^+^-ATPase and interaction with the 14-3-3 proteins (5) were enhanced by the application of Mg and/or IAA (6) under Al stress, resulting in activation of both the plasma membrane H^+^-ATPase and the MATE citrate transporter. Citrate exuded from plant roots forms a stable non-toxic complex with Al in the rhizosphere (7).

Electrophysiological analysis using *Xenopus oocytes* showed that the MATE family transporters from sorghum (SbMATE) (Magalhaes et al., 2007), maize (ZmMATE1) (Maron et al., 2010), and rice bean (VuMATE1) (Yang et al., 2011c) mediated significant ^14^C-labeled-citrate efflux as well as proton influx across the plasma membrane, suggesting that the MATEs might be citrate/H^+^ antiport transporters (Kochian et al., 2015). This suggestion, however, needs to be interpreted with respect to the ionic as well as charge gradients across the plasma membrane. Citric acid is a triprotic weak acid having pKa values for the three stepwise carboxylic group dissociations of 3.1, 4.8, and 6.4. Hence, at a relatively high (∼7.0) cytosolic pH, citric acid is dissociated to citrate anion (Cit^3-^) and H^+^ (**Figure 2**). The MATE-mediated citrate anion efflux is likely to be coupled with the H^+^-ATPase-driven H^+^ efflux (to balance charges), meaning that Al-induced citrate exudation is mediated by a plasma membrane co-transport (symport) system in which H^+^-ATPase and MATE are coupled. The H^+^ influx (purportedly via MATE transporters) as observed in *Xenopus* oocytes (Magalhaes et al., 2007; Maron et al., 2010; Yang et al., 2011c) may be secondary in nature, and its role remains to be properly explained. It may be tempting to speculate that such influx of H^+^ may be associated with the general maintenance of cytosolic pH and the plasma membrane gradients as well as balancing of secondary ion transports rather than primarily as an antiport action directly coupled with citrate exudation via MATEs. This explanation is well aligned with the primary roles of MATEs as organic cation (citrate is never a cation) antiporters (coupled with H^+^ or Na^+^ influx, Jin et al., 2014) in waste disposal (e.g., in kidney and liver cells, Nies et al., 2016) as well as in bacterial or cancer cell resistance to drugs (e.g., Tanaka et al., 2013). Hence, there is no doubt that MATEs can allow H^+^ influx (for the mechanism, see Nishima et al., 2016), but we would suggest it is premature to assign the label of citrate/H^+^ antiporters to the MATE transporters functioning in citrate (anion) efflux. Alternatively, it may also be tempting to suggest that in plants MATEs transport Al::citrate complexes from roots to shoots, thus playing a role in Al detoxification, rather than effluxing citrate from roots to mediate Al exclusion at the root surface; however, there is no experimental evidence to support this suggestion, except that MATE transporter OsFRDL1 was found to transport Fe::citrate complexes from rice roots to shoots (Yokosho et al., 2009), and the same route of Al::malate transport in *Arabidopsis* was claimed for the nodulin 26-like intrinsic protein (NIP1;2) from the aquaporin family (Wang J. et al., 2017; Wang Y. et al., 2017). The MATE transport system in plants is indeed complex, and a substantial future work is needed to shed more light on that complexity.

## Activation of the Plasma Membrane H^+^-ATPase Improves Plant Resistance to Aluminum Stress

### Magnesium Availability

Magnesium (Mg) is an essential nutrient for plant growth and has a number of key functions in plants (Cakmak and Kirkby, 2008). It is an activator of a large number of enzymes, such as phosphatases, protein kinases, RNA polymerases, and carboxylases (Bose et al., 2011). Magnesium is also pivotal to the function of most ATPase proteins and essential for maintaining the proton pump activity at the plasma membrane (Brooker and Slayman, 1983; Gibrat et al., 1989). Under constant Al^3+^ ionic activity, the Al-resistant genotypes had higher Mg concentration than the Al-sensitive genotypes of wheat (Silva et al., 2010), maize (Mariano and Keltjens, 2005; Giannakoula et al., 2008), sorghum (Baligar et al., 1993), *Arabidopsis* (Bose et al., 2013), and rice (Sivaguru and Paliwal, 1993). Additionally, it has been shown that Mg can alleviate Al toxicity to plants (Rengel et al., 2015). Relatively high concentrations (millimolar) of Mg were found to alleviate Al toxicity in Poaceae species, such as wheat and rice (Ryan et al., 1997; Watanabe and Okada, 2005; Chen Z.C. et al., 2012). This effect may be attributed to Mg reducing Al saturation at the apoplasmic binding sites and decreasing Al activity at the root-cell plasma-membrane surface. On the other hand, in legumes, relatively low (micromolar) concentrations of Mg significantly alleviated Al-induced root growth inhibition in soybean (Silva et al., 2001), rice bean (Yang et al., 2007), and faba bean (Chen et al., 2015) through enhancement of Al-induced citrate exudation. It is important to point out that the Mg-related enhancement of Al-induced citrate exudation was dependent on the activation of the plasma membrane H^+^-ATPase in rice bean (Yang et al., 2007) and faba bean (Chen et al., 2015).

Chen et al. (2015) determined the expression of the genes encoding MATE as well as the plasma membrane H^+^-ATPase in the presence or absence of Mg under Al stress in faba bean roots. The expression of both a putative *MATE*-like gene and *vha2* (*Vicia faba* plasma membrane H^+^-ATPase 2) was induced by Al but not Mg. Furthermore, immunoprecipitation, pull-down and Western blot analyses showed that phosphorylation of the penultimate Thr-residue in VHA2, as well as its *in vivo* and *in vitro* association with the vf14-3-3b protein, both increased in the presence of Mg under Al stress, which corresponded well with the Mg-mediated enhancement of (i) the plasma membrane H^+^-ATPase activity and (ii) citrate exudation under Al stress (Chen, 2012; Chen et al., 2015). The Mg-mediated increase in phosphorylation of the Thr-residue in the plasma membrane H^+^-ATPase may enhance binding of the 14-3-3 protein and thus activation of the enzyme.

### Auxin Transport

The auxin signaling has been shown to be involved in abiotic stresses such as salt (Albacete et al., 2008) and low P (Shen et al., 2006). In soybean roots, low-P induced endogenous IAA accumulation that (similarly to an exogenous application of 10 μM IAA) increased the activity of plasma membrane H^+^-ATPase and enhanced P uptake (Shen et al., 2006). In *Arabidopsis*, the *pin2* and *aux1* mutants exhibited the much decreased activity of the plasma membrane H^+^-ATPase and depressed root elongation under alkaline (Xu et al., 2012) and acidic stress (Inoue et al., 2016).

The Al-induced auxin accumulation in *Arabidopsis* root-apex transition zone was crucial for Al-related root growth inhibition (Yang et al., 2014). Primary root elongation was less inhibited in the *Arabidopsi*s auxin-polar-transport mutants *AUX1* (AUXIN RESISTANT 1) and *PIN2* (PIN-FORMED) than wild-type plants under Al stress (Sun et al., 2010). Consistently, auxin signaling was involved in the Al-induced primary root growth inhibition, but promoted lateral root formation and maturation (Ruiz-Herrera and Lopez-Bucio, 2013). In wheat roots, the Al-induced endogenous IAA accumulation correlated significantly with malate exudation; similarly, the exogenous treatment with 10 μM IAA enhanced, whereas that with 30 μM 1-naphthylphthalamic acid (NPA, an auxin efflux transport inhibitor) decreased, malate efflux and Al concentration (Yang et al., 2011d). In soybean roots, Wang (2014) found that the mRNA abundance of *GmPIN2* and IAA concentration in soybean roots were increased after treatment with 25 or 50 μM Al, but not 200 μM Al. Furthermore, the Al-induced citrate exudation as well as the expression of *GmMATE* and the phosphorylation of the plasma membrane H^+^-ATPase in soybean roots were significantly increased by IAA, but decreased by IAA transport inhibitor TIBA (2,3,5-triiodobenzoic acid) (Wang et al., 2016). In accordance with these pharmacological results obtained in soybean, the genetic data using *pin2* and *aux1-7 Arabidopsis* mutants showed a decrease not just in the activity of the plasma membrane H^+^-ATPase, but also in citrate exudation and expression of *AtMATE* (Yu, 2017). Hence, it is suggested that activation of the plasma membrane H^+^-ATPase and upregulation of the *MATE* expression are involved in the auxin enhancement of Al-induced citrate exudation.

### Transgenic Methods to Increase Activity of the Plasma Membrane H^+^-ATPase

Overexpressing either the plasma membrane H^+^-ATPase directly or its regulatory components has been shown to be effective in enhancing the activity of this enzyme. Ectopic expression of *PeHA1* from *Populus euphratica* significantly increased the proton pumping activity of the plasma membrane H^+^-ATPase and salt tolerance in transgenic *Arabidopsis* (Wang et al., 2013). In tobacco plants, expressing a plasma membrane H^+^-ATPase isoform lacking the autoinhibitory domain (ΔPMA4) constitutively activated the plasma membrane H^+^-ATPase and resulted in abnormal leaf inclination and twisted stems, suggesting alterations in cell expansion (Gevaudant et al., 2007). Under salt stress conditions, the ΔPMA4 plants displayed increased salt tolerance during germination and seedling growth (Gevaudant et al., 2007).

Under Al stress, overexpression of AHA1 in *Arabidopsis* significantly increased organic acid anion exudation and Al resistance in comparison with the wild-type plants (Wu et al., 2008). To investigate the interaction between 14-3-3 proteins and plasma membrane H^+^-ATPase of soybean in the regulation of citrate exudation and Al resistance, Guo (2013) obtained transgenic tobacco overexpressing soybean *SGF14a* that encodes a 14-3-3 protein. The expression of *SGF14a* in tobacco significantly increased the activity and phosphorylation of the plasma membrane H^+^-ATPase and interaction with the 14-3-3 proteins, resulting in activation of the Al-induced citrate exudation and Al resistance. Overexpression of soybean Δ*GHA2* lacking the autoinhibitory domain of the plasma membrane H^+^-ATPase isoform had no effect on phosphorylation of the plasma membrane H^+^-ATPase and the interaction with 14-3-3 proteins, but it significantly improved plasma membrane H^+^-ATPase activity, citrate exudation, Al resistance, and the growth of transgenic tobacco in acidic soil (Guo, 2013).

## Conclusion

The plant plasma membrane H^+^-ATPase plays the important roles in plant growth under optimal and Al stress conditions. In several plant species, the Al-resistant genotypes show higher activity of the plasma membrane H^+^-ATPase than the Al-sensitive ones, which is associated with higher gene expression and protein abundance as well as the penultimate threonine-residue phosphorylation of the enzyme and its interaction with 14-3-3 proteins. Providing the motive force for MATE-mediated transport of citrate anion out of root epidermal cells and maintaining the physiological H^+^ and charge gradients across the plasma membrane might be the two main functions of the plasma membrane H^+^-ATPase under Al stress. Further work is needed to improve plant Al resistance by modulating activity of the plasma membrane H^+^-ATPase, especially in plant species relying on facilitated citrate exudation as the main Al-resistance mechanism. However, some fundamental questions remain unclear. For example, activation of the plasma membrane H^+^-ATPase occurs also under P deficiency and salt and heavy metal stresses. Possibly, Al toxicity shares a similar signaling pathway with these abiotic stresses to regulate the plasma membrane H^+^-ATPase activity. Moreover, the research on the interaction between Al toxicity and plasma membrane H^+^-ATPase has focused so far on just several plant species, such as soybean, faba bean, wheat, tea, and *Arabidopsis*; therefore, the function of plasma membrane H^+^-ATPase in response to Al toxicity in other plant species, especially in Gramineae such as barley, maize, and sorghum where MATE-mediated citrate exudation occurs under Al stress, needs to be elucidated.

## Author Contributions

ZR and QC conceived this work, wrote and revised the manuscript. JZ, JW, and DL co-wrote the manuscript. XK, LC, YY, and XC provided suggestions and revised the manuscript. All of the authors reviewed the final version of the manuscript and approved the manuscript for publication.

## Conflict of Interest Statement

The authors declare that the research was conducted in the absence of any commercial or financial relationships that could be construed as a potential conflict of interest.
